# Sin nombre virus (SNV) Ig isotype antibody response during acute and convalescent phases of hantavirus pulmonary syndrome.

**DOI:** 10.3201/eid0602.000213

**Published:** 2000

**Authors:** P Bostik, J Winter, T G Ksiazek, P E Rollin, F Villinger, S R Zaki, C J Peters, A A Ansari

**Affiliations:** Emory University School of Medicine, Atlanta, GA 30322, USA.

## Abstract

Serum samples from 22 hantavirus pulmonary syndrome (HPS) patients were tested for Sin Nombre virus (SNV)-reactive antibodies. In the acute phase of HPS, 100% and 67% of the samples tested positive for SNV-specific immunoglobulin (Ig) M and IgA, respectively. Among the virus-specific IgG antibodies, the most prevalent were IgG3 (in 97% of samples), followed by IgG1 (70%), IgG2 (30%), and IgG4 (3%).


### Sin Nombre Virus (SNV) Ig Isotype Antibody Response during Acute and Convalescent Phases of Hantavirus Pulmonary Syndrome

**Pavel Bostik,* Jorn Winter,*† Thomas G. Ksiazek,† Pierre E. Rollin,† Francois Villinger,* Sherif R. Zaki,† C.J. Peters,† and Aftab A. Ansari***
*Emory University School of Medicine, Atlanta, Georgia, USA; and †Centers for Disease Control and Prevention, Atlanta, Georgia, USA

Serum samples from 22 hantavirus pulmonary syndrome (HPS) patients were tested for Sin Nombre virus (SNV)-reactive antibodies. In the acute phase of HPS, 100% and 67% of the samples tested positive for SNV-specific immunoglobulin (Ig) M and IgA, respectively. Among the virus-specific IgG antibodies, the most prevalent were IgG3 (in 97% of samples), followed by IgG1 (70%), IgG2 (30%), and IgG4 (3%).

Hantaviruses are associated with hemorrhagic fever with renal syndrome (HFRS) and hantavirus pulmonary syndrome (HPS) in humans (1). Sin Nombre virus (SNV), a newly identified hantavirus, was identified as the causative agent in a 1993 outbreak of HPS with a >60% case-fatality rate in the southwestern United States (2). SNV and other American hantaviruses associated with HPS have a specific rodent reservoir; transmission was inferred to occur primarily by inhalation of infected aerosols from the rodent urine and excreta (3,4). However, an outbreak with human-to-human transmission of HPS caused by Andes virus has also been reported from South America (5). HPS is clinically characterized as an acute febrile illness associated with headache, malaise, and myalgia that proceeds to thrombocytopenia, pulmonary edema, and hypotension or shock (1,2). In fatal cases, death usually occurs 1 to 2 days after onset of respiratory symptoms. Although SNV-specific $CD4^+$ and $CD8^+$ T lymphocytes have been documented (6), the virus-specific humoral immune response has not yet been well defined.

The different patterns of increase in immunoglobulin (Ig) classes are associated with Th1- or Th2-type immune responses in mice (7). In some parasitic diseases, increased antigen-specific IgG4 subclass levels correspond to a variable extent with the induction of antigen-specific T-cell anergy, sometimes associated with serious or disseminated disease (8,9). Antibody responses to polysaccharide antigens have been reported to be predominantly IgG1/IgG2 (10). In viral diseases, IgG1 and IgG3 subclasses were detected predominantly in primary infections, while IgG2 and IgG4 subclasses were more characteristic of recurrent infections (11-14).

Ig class- and subclass-specific titers of antibodies to SNV, their kinetic appearance, and their role in preventing disease or death in HPS patients has not been reported. To address this issue, we developed SNV-specific, immunoglobulin class/subclass-specific enzyme-linked immunosorbent assays and evaluated the levels of SNV-specific IgA, IgM, and IgG subclass antibodies in the serum samples from HPS patients. Ig class- and subclass-specific titers were compared in the sera of patients who survived and those who died.

### The Study

Thirty-three serum samples were obtained from 22 patients hospitalized with HPS in 1993. Patients were diagnosed as having HPS on the basis of results from immunohistochemistry

Address for correspondence: A.A. Ansari, Winship Cancer Center, Emory University School of Medicine, 1365B Clifton Rd, Atlanta, GA 30322, USA; fax: 404-778-5016; e-mail: pathaaa@emory.edu.

analysis, alone or in combination with serologic studies or reverse transcription-polymerase chain reaction (16). The samples were divided into three groups with respect to the time of collection: acute phase (1-8 days posthospitalization, 12 samples), early convalescent phase (9-30 days posthospitalization, 9 samples), and late convalescent phase (31 days to 1.5 years posthospitalization, 12 samples).

Human Ig antibodies were captured by affinity-purified, polyclonal, monospecific sheep antihuman IgM, IgG1, IgG2, IgG3, IgG4, and IgA reactive antibodies (IgM: Biosource, Camarillo, CA; IgG1, IgG2, IgG3, IgG4, IgA: The Binding Site, San Diego, CA). The capture antibodies were coated onto 96-well high-binding microtiter plates (Corning Costar Corporation, Charlotte, NC) at 4°C for a minimum of 16 hours (0.5 mg/ml in 25mM sodium carbonate buffer pH 9.6; 0.1 ml/well). Sera and detection antibodies were diluted in PBS, pH 7.4 to 7.6, containing 0.05% Tween 20 supplemented with 4% milk (PBSM). Initially, all plates were washed and blocked with PBSM for 30 minutes at room temperature. Fourfold dilutions of the plasma samples in a volume of 0.1 ml were incubated for 1 hour at 37°C, then the plates were washed and SNV-infected Vero E6 cell slurries (CDC, Atlanta, GA; SPR 268) and uninfected control slurries (CDC, Atlanta, GA; SPR 391), diluted 1:10, were added to appropriate wells. After incubation for 1 hour at 37°C, the plates were washed and incubated with a polyclonal rabbit anti-SNV serum (CDC, Atlanta, GA; diluted 1:1000, 0.1 ml/well) as described. Then the plates were washed and an alkaline phosphatase-conjugated sheep Ig anti-rabbit Ig (Southern Biotechnology Associates, Birmingham, AL) diluted 1:4000 (0.1 ml/well) was added to each well. After incubation for 1 hour at 37°C, the plates were washed and the substrate p-nitrophenylphosphate (Bio-Rad, Hercules, CA) was added. The reaction was stopped by the addition of 0.1 ml 0.4 M NaOH, and the optical density was read at 405 nm.

Where applicable, antibody titers are presented as geometric mean titers (GMT), with the range of positive titers. Antibody titers for significant differences between survivors and patients who died in the acute-phase group were compared by unpaired Student's t-test.

SNV-specific IgM, which had the highest titers overall (GMT 15,097, range 1,600-102,400), was detected in all acute-phase samples and 89% of samples from the early convalescent phase (Table). SNV-specific IgM was also detected in one sample from the late convalescent phase (titer 25,600 at day 67), a phenomenon occasionally observed in other HPS convalescent-phase samples (data not shown). SNV-specific serum IgA was detected in 67% of acute-phase samples and 78% of samples from the early convalescent phase, but the titers were generally lower (GMT 9,406, range 100 to 409,600) than the IgM titers. Two of the 12 patients during the late convalescent phase had detectable virus-specific IgA, although the titers were relatively low (1,600 and 100). Patients who had very low or undetectable SNV-specific IgA generally also had lower titers of SNV-specific IgM during the acute phase of illness.

In the IgG class, the highest SNV-specific antibody titers were predominantly of the IgG3 subclass (GMT 4,935, range 100-409,600), detected in 97% of samples. SNV-specific IgG1 antibodies were also detected in 76% of samples, but in much lower titers (GMT 447, range 100-6,400). SNV-specific IgG2 antibodies were detected in only 30% of samples (GMT 348, range 100-6,400), and only one serum tested positive for SNV-specific IgG4, with a very low titer (1:100).

The Ig class- and subclass-specific antibody titers were further analyzed with regard to the outcome of the disease. The 12 patients who had samples drawn during the acute phase of infection included six who died and six who survived. The SNV-specific antibody titers of these two groups did not differ significantly in any Ig class or subclass tested ($p > 0.1$ in each Ig class or IgG subclass group, data not shown).

When total IgG, IgA, and IgM concentrations were measured in 18 serum samples from randomly chosen HPS patients, 12% of the IgM values (reference range 56-332 mg/dl), 39% of the IgA values (reference range 70-312 mg/dl) and 56% of the IgG values (reference range 639-1349 mg/dl) fell outside the normal reference range. Interestingly, increased titers of both SNV-specific IgA and IgM did not correlate with patients' levels of total serum IgA and IgM. This lack of correlation suggests that the increase of SNV-specific IgM and IgA antibodies is not due to nonspecific polyclonal activation of the immune system.

As expected, SNV-specific IgM antibodies are observed during the initial phase of disease; both SNV-specific serum IgA and IgM antibodies peaked early during the acute phase. These levels were

Table. Total Ig concentrations and specific Sin Nombre virus Ig titers in sera from patients with hantavirus pulmonary syndrome

| Patient[a] | Days[b] | IgM | IgG1 | IgG2 | IgG3 | IgG4 | IgA | Total IgM [mg/dl] | Total IgG [mg/dl] | Total IgA [mg/dl] | Phase |
|---|---|---|---|---|---|---|---|---|---|---|---|
| 7(d)[c] | 1 | 25,600 | 400 | 0 | 1,600 | 0 | 25,600 | 264 | 1,000 | 336 | Acute |
| 8(d) | 1 | 1,600 | 0 | 0 | 100 | 0 | 0 | 559 | <333 | <67 | Acute |
| 9(d) | 1 | 25,600 | 400 | 0 | 6,400 | 0 | 102,400 | 295 | 802 | <40 | Acute |
| 6.1 | 2 | 102,400 | 6,400 | 100 | 25,600 | 0 | 25,600 | 280 | 1,590 | 492 | Acute |
| 10(d) | 2 | 25,600 | 100 | 0 | 400 | 0 | 0 | nd[d] | 4,840 | 234 | Acute |
| 5.1 | 2 | 25,600 | 100 | 0 | 1,600 | 0 | 409,600 | 1,080 | 176 | 342 | Acute |
| 21.1 | 4 | 102,400 | 100 | 6,400 | 409,600 | 0 | 0 | 409,600 | nd | nd | Acute |
| 23.1 | 7 | 25,600 | 100 | 100 | 400 | 0 | 25,600 | nd | nd | nd | Acute |
| 1.1 | 7 | 1,600 | 400 | 100 | 6,400 | 0 | 400 | 142 | 401 | 108 | Acute |
| 11(d) | 7 | 102,400 | 100 | 0 | 400 | 0 | 6,400 | nd | nd | nd | Acute |
| 23.2 | 8 | 25,600 | 0 | 0 | 100 | 0 | 0 | nd | nd | nd | Acute |
| 12(d) | 8 | 6,400 | 0 | 0 | 0 | 0 | 0 | 108 | <381 | <67 | Acute |
| 4.3 | 10 | 6,400 | 0 | 0 | 1,600 | 0 | 25,600 | nd | nd | nd | Early conv.[e] |
| 2.1 | 12 | 25,600 | 6,400 | 1,600 | 25,600 | 0 | 409,600 | 225 | 508 | 125 | Early conv. |
| 21.2 | 13 | 102,400 | 100 | 6,400 | 409,600 | 100 | 409,600 | nd | nd | nd | Early conv. |
| 23.3 | 13 | 6,400 | 0 | 0 | 100 | 0 | 100 | nd | nd | nd | Early conv. |
| 3.1 | 16 | 6,400 | 6,400 | 100 | 6,400 | 0 | 0 | 322 | 1,170 | 290 | Early conv. |
| 1.2 | 21 | 25,600 | 6,400 | 400 | 25,600 | 0 | 0 | nd | nd | nd | Early conv. |
| 4.1 | 24 | 1,600 | 0 | 0 | 6,400 | 0 | 6,400 | nd | nd | nd | Early conv. |
| 13 | 24 | 0 | 6,400 | 0 | 102,400 | 0 | 102,400 | nd | nd | nd | Early conv. |
| 14 | 30 | 1,600 | 0 | 0 | 1,600 | 0 | 100 | nd | nd | nd | Early conv. |
| 15 | 34 | 0 | 100 | 0 | 6,400 | 0 | 0 | 201 | 1,400 | 263 | Late conv. |
| 3.2 | 35 | 0 | 6,400 | 0 | 6,400 | 0 | 0 | 176 | 2,110 | 221 | Late conv. |
| 4.2 | 59 | 0 | 0 | 0 | 1,600 | 0 | 1,600 | 144 | 1,310 | 95 | Late conv. |
| 2.2 | 66 | 0 | 1,600 | 100 | 6,400 | 0 | 0 | 126 | 1,140 | 140 | Late conv. |
| 16 | 66 | 0 | 100 | 0 | 25,600 | 0 | 0 | 69 | 977 | 82 | Late conv. |
| 20.2 | 67 | 0 | 400 | 100 | 6,400 | 0 | 100 | nd | nd | nd | Late conv. |
| 6.2 | 67 | 25,600 | 6,400 | 0 | 102,400 | 0 | 100 | 219 | 1,700 | 403 | Late conv. |
| 17.1 | 92 | 0 | 100 | 0 | 102,400 | 0 | 0 | 107 | 1,000 | 162 | Late conv. |
| 5.2 | 145 | 0 | 100 | 0 | 1,600 | 0 | 0 | 78 | 1,260 | 288 | Late conv. |
| 18 | 330 | 0 | 100 | 0 | 25,600 | 0 | 0 | nd | nd | nd | Late conv. |
| 17.2 | 330 | 0 | 100 | 0 | 6,400 | 0 | 0 | nd | nd | nd | Late conv. |
| 19 | 547 | 0 | 100 | 0 | 400 | 0 | 0 | nd | nd | nd | Late conv. |

[a]Decimal values indicate sequential samples from one patient.
[b]Days after patient reported to hospital.
[c](d) = patient died.
[d]nd = not done.
[e]conv. = convalescent.

maintained during both the acute and the early convalescent phases. SNV-specific antibodies of IgG1 and IgG3 subclasses appeared during the first 10 days, coinciding with the appearance of symptoms. However, high titers were maintained throughout early (IgG1) or early and late convalescent phases (IgG3). In 5 (70%) of the 7 samples that tested positive for SNV-specific IgG2 antibodies, these titers appeared during the acute and early convalescent phases but were lost thereafter.

### Conclusions

We tested serum and plasma samples from HPS patients for the presence of IgA and IgM class and the IgG subclass-specific antibody titers against one of the SNV antigens. Although the design of this study is cross sectional, we observed a characteristic pattern of appearance of specific Ig classes/subclasses during the course of infection. In addition, sequential samples from several patients were included in the study (represented by the decimal values in the Table)

and the data from these samples show trends similar to those observed in the composite with single samples. SNV-specific IgM and IgA antibodies appeared in high titers predominantly during the early phases of infection (in 95% and 71% of samples, respectively). In our IgG subclass-specific assays, most samples contained IgG3 and IgG1 subclass-specific SNV-reactive titers throughout illness. The detection of substantial IgG3 antibody titers early in hospitalization can be explained by the fact that since infection occurred several days before onset of illness, there was sufficient time to allow a switch to the IgG class to occur. In contrast with other primary viral infections, a substantial number of samples showed high titers of SNV antigen-reactive antibodies belonging to the IgG3 subclass rather than the IgG1 subclass. It is possible that in SNV infection, specific sets of cytokines may be produced that preferentially stimulate the production of IgG3 subclass antibody.

The similarity in antibody titers between deceased and surviving patients is an important observation. Regardless of the eventual outcome of infection, the sera from patients during hospitalization with either HPS or HFRS contained high levels of antibody. Abundant viral antigens were present within endothelial cells of the pulmonary microvasculature, as well as significant levels of $CD8^+$ T-cell infiltrates and evidence of circulating pro-inflammatory cytokines in serum. These findings suggest that the disease is most likely secondary to immunopathologic mechanisms (6,15,16). Thus, antibodies may well play an important role in containing the initial viremic phase of the infection, but T-cell activation may have an important role in inducing disease. In addition, there may be epitopes of the virus antigens that are not detected by the assays used in the studies reported here and antibodies against these epitopes may distinguish infected persons who do or do not become ill. Further studies to identify differences in the viral epitopes recognized by sera from patients who died compared with those who survived, as well as comparison of antibody functionality, are needed to address these issues. Finally, anti-SNV IgM antibodies were detected in 100% of the patients in the acute phase of the infection in relatively high titers (1,600 to 102,400). This finding confirms that the SNV-specific IgM-capture enzyme immunoassay we describe can be used as a valuable tool in the early diagnosis of HPS.

### Acknowledgments

The authors thank the patients, health-care providers, and local and state health departments, who have been crucial for the collection of the samples.

This work was supported by the Centers for Disease Control and Prevention cooperative agreement # U50/ CCU411374-03-01.

Dr. Bostik is an instructor of pathology in the Department of Pathology and Laboratory Medicine, Emory University School of Medicine, Atlanta, GA. His interests focus on molecular immunology and T-cell immunity in humans and nonhuman primate models of viral infections.

### References

1. Peters CJ, Simpson GL, Levy H. Spectrum of hantavirus infection: hemorrhagic fever with renal syndrome and hantavirus pulmonary syndrome. Annu Rev Med 1999;50:531-45.
2. Duchin JS, Koster FT, Peters CJ, Simpson GL, Tempest B, Zaki SR, et al. Hantavirus pulmonary syndrome; clinical description of disease caused by a newly recognized hemorrhagic fever virus in the southwestern United States. N Engl J Med 1994;330:949-55.
3. Peters CJ. Hantavirus pulmonary syndrome in the Americas. In: Scheld WM, Craig WA, Hughes JM, editors. Emerging Infections II. Washington: American Society for Microbiology Press; 1998. p. 17-64.
4. Zeitz PS, Butler JC, Cheek JE, Samuel MC, Childs JE, Shands LA, et al. A case-control study of hantavirus pulmonary syndrome during an outbreak in the southwestern United States. J Infect Dis 1995;171:864-70.
5. Padula PJ, Edelstein A, Miguel SD, Lopez NM, Rossi CM, Rabinovich RD. Hantavirus pulmonary syndrome outbreak in Argentina: molecular evidence for person-to-person transmission of Andes virus. Virology 1998;241:323-30.
6. Ennis FA, Cruz J, Siropoulou CF, Waite D, Peters CJ, Nichol ST, et al. Hantavirus pulmonary syndrome: CD8+ and CD4+ cytotoxic T lymphocytes to epitopes on Sin Nombre virus nucleocapsid protein isolated during acute illness. Virology 1997;238:380-90.
7. Snapper CM, Paul WE. Interferon-gamma and B cell stimulatory factor-1 reciprocally regulate Ig isotype production. Science 1987;236:944-7.
8. Yazdanbakhsh M, Paxton WA, Kruize YCM, Sartono E, Kurniawan A, van het Wout A, et al. T cell responsiveness correlates differentially with antibody isotype levels in clinical and asymptomatic filariasis. J Infect Dis 1993;167:925-31.
9. Ulrich M, Rodriguez V, Centeno M, Convit J. Differing antibody IgG isotypes in the polar forms of leprosy and cutaneous leishmaniasis characterized by antigen-specific T cell anergy. Clin Exp Immunol 1995;100:54-8.
10. Rautonen N, Pelkonen J, Sipipnen S, Kayhty H, Makela O. Isotype concentrations of human antibodies to group A meningococcal polysaccharide. J Immunol 1986;137:2670-5.

11. Hashido M, Kawana T. Herpes simplex virus-specific IgM, IgA and IgG subclass antibody responses in primary and nonprimary genital herpes patients. Microbiol Immunol 1997;41:415-20.
12. Wagner DK, Muelenaer P, Henderson FW, Snyder MH, Reimer CB, Walsh EE, et al. Serum immunoglobulin G antibody subclass response to respiratory syncytial virus F and G glycoproteins after first, second, and third infections. J Clin Microbiol 1989;27:589-92.
13. Torfason EG, Reimer CB, Keyserling HL. Subclass restriction of human enterovirus antibodies. J Clin Microbiol 1987;25:1376-9.
14. Forthal DN, Landucci G, Habis A, Zartarian M, Katz J, Tilles JG. Measles virus-specific functional antibody responses and viremia during acute measles. J Infect Dis 1994;169:1377-80.
15. Ksiazek TG, Peters CJ, Rollin PE, Zaki SR, Nichol S, Spiropoulou C, et al. Identification of a new North American hantavirus that causes acute pulmonary insufficiency. Am J Trop Med Hyg 1995;52:117-23.
16. Zaki SR, Greer PW, Coffield LM, Goldsmith CS, Nolte KB, Foucar K, et al. Hantavirus pulmonary syndrome: pathogenesis of an emerging infectious disease. Am J Pathol 1995;146:552-79.
